# Missing data as data

**DOI:** 10.1016/j.patter.2022.100587

**Published:** 2022-09-09

**Authors:** Anahid Basiri, Chris Brunsdon

**Affiliations:** 1 School of Geographical and Earth Sciences, The University of Glasgow, Glasgow, G12 8QQ Glasgow, UK; 2 National Centre for Geocomputation, National University of Ireland, Maynooth, Ireland

**Keywords:** missing data, big data paradox, under-representation, bias, crowdsourced data

## Abstract

Our “digified” lives have provided researchers with an unprecedented opportunity to study society at a much higher frequency and granularity. Such data can have a large sample size but can be sparse, biased, and exclusively contributed by the users of the technologies. We look at the increasing importance of missing data and under-representation and propose a new perspective that considers missing data as useful data to understand the underlying reasons for missingness and that provides a realistic view of the sample size of large but under-represented data.

## Main text

### Democracy is not the autocracy of majority on the minority

Over the last few decades, the popularity of social media platforms technologies have given scientists an unprecedented opportunity to study and monitor society and the physical world with potentially near-population size data updating at a higher frequency than traditional surveys and polls, often at a much lower cost, on some occasions free. These datasets could give an impression that we can have a good understanding of the whole population. However, they rely on the willingness of volunteers to share, contribute, respond, or report. Self-reported and crowdsourced data can only reflect some groups of individuals or cases, or some sections of society, while others can be omitted or at least under-represented. With crowdsourced data, the possibility of repetition of the same view from the same type of people may give the impression of a larger agreement. Also, when significant numbers of people are discussing a sensitive topic such as elections on platforms such as Twitter, the ones who disagree with the majority may be reluctant to share their views. This does not mean that almost the entire population agree on a topic, it simply means that those who use the platform and feel free to express their views are in agreement. Therefore, any services or decisions that arise from such volunteered data—even if they constitute the majority of the population—can overlook the needs of non-respondents. This can ultimately reinforce the isolation of such groups, as they may not be able to use a new service or receive much-needed resources, and so their voices remain even more unheard. The amplified bias in the data-driven approach can only be avoided if we know who is missing and their representatives have shared their views.

In addition to the distorted view of society, the stand-alone use of new forms of data can lead to unethical decisions. Many of the services we use daily are not designed to serve only the majority. In many cases, the majority is flexible and open to several options, while there is a small minority that cannot tolerate some of the options or the impacts of some options are significantly high. For example, we have accessible buildings and public transport not because they accommodate the needs of the majority but because there is a minority whose social, economic, and personal life and mobility depend on accessibility. Relying on self-reported or volunteered data, with no sampling strategy implemented, can result in not hearing the voice of the most affected minority groups at all, even if the size of the sample is more than 50% of the population.

### No comment can be a comment

Missing users and their data are increasingly important when analyzing volunteered information, self-reported data, or an observational byproduct of digitized transactions.^1^ However designed surveys also suffer through declining response rates. These can have many reasons, including lack of interest or availability, privacy concerns, or other personal reasons to remain silent. If the missing values are not at random, then one can assume there is a reason for missingness that can potentially be linked to the missing values. Ideally, datasets used for research are the outcome of rigorous research design. However, many datasets now simply rely on the individuals’ readiness to share, contribute, or respond—in some cases unknowingly.

Missing users, where a person refuses to respond to *any* question in a survey, or missing values, where a participant has provided a partial response (i.e., replied to some questions but not all), have been an issue in traditional surveys, too.^2^ However, self-reported data can have missing values on different scales and levels. “Missing Not at Random” is common in such data, e.g., where a participant has answered questions or shared data with the exception of some, potentially with a sensitive outcome, e.g. their level of income, where a “prefer not to answer” can be recorded. One can explore whether the propensity to respond is linked to the unrecorded value and find the relationship between the missingness and missing value, which may explain the underlying reasons behind the unavailability of data. This can help the design of more inclusive platforms and surveys that address their concerns.

### Effective sample size of big data

The crowdsourcing platforms are *technically* open to all, but still many cannot or do not wish to use them. Not everybody has a representative to express their view, and some views are over-represented due to several cognitive biases such as confirmation bias (see for example Pang et al.^3^ and self-serving bias^4^). A designed survey takes greater care to overcome potential issues such as these, but available resources generally dictate that the number of individuals included in a sample is considerably smaller. Thus, although the bias is smaller, the sampling error is greater. However, overall error (measured by the mean square error, or MSE) is a combination of both sources and can be estimated given a sample size and a model of bias. In this regard, measuring what is the equivalent MSE for a “big data” source and the results of a randomized sample is very important to have a better understanding of big data “quality-quantity paradox.”^5^

The *effective sample size* describes the size of a sample (obtained by simple random sampling) from a population of size *N*, whose sample mean is as accurate an estimator of the population mean in terms of MSE as that of a sample of size *n*_*R*_ acquired by some non-random sampling procedure.^5^ In particular, the effective sample size for PCR testing data can be written:

(Equation 1) $$n_{eff}=\frac{n_{R}}{f_{R}+\left(1-f_{R}\right)\left(N-1\right)E_{R}\left[\rho_{R,g}^{2}\right]}\approx \frac{f_{R}}{1-f_{R}}\frac{1}{\rho_{R,g}^{2}},$$

where $n_{eff}$ is the effective sample size of a self-reporting dataset, $\frac{f}{1-f}$ is the “drop-out” measure that is an indicator of participation of population, *N* is the population size, and *E* is the data defect index.^5^

Once the sample is considered to be randomized, and we have accounted for distortion due to bias, we arrive at the *big data paradox* where we are bound by the interplay between the three elements of data quality, problem difficulty, and sample size (see Equation 2), due to:^5^

(Equation 2) $${\hat{\mu }}_{g}-\mu_{g}=\underset{\mathrm{Data}\mathrm{Quality}}{\underset{︸}{\rho_{R,g}}}\times \underset{\mathrm{Problem}\mathrm{Difficulty}}{\underset{︸}{\sigma_{g}}}\times \underset{\mathrm{Data}\mathrm{Quantity}}{\underset{︸}{\sqrt{\frac{N-n}{n}}}}$$

where the difference between the mean of the sample $\left({\hat{\mu }}_{g}\right)$ and the true mean of the population $\left(\mu_{g}\right)$ on the left side of the equation is calculated by the data quality, problem difficulty, and the number of the participants or self-reporting people. The first element, data quality, is the correlation between the responding behavior and the value. In a stratified randomized survey, this should be zero as we select individual regardless of their responses to the questions. However, in self-reported data, there can be a correlation between the missing value and missingness, e.g., not declaring income because it is too high. This correlation is difficult to calculate, unlike the data quantity and the problem difficulty, i.e. standard deviation of the target value. One approach to this could be recognizing the patterns of missingness in different datasets in the same area. For example, to see whether residents of certain neighborhood with higher average of income are more likely to “prefer not to answer” the questions about income. As we explain in the next section, this correlation, of course, does not mean causation, and also zero correlation does not mean well representation.

### Zero does not always mean zero

A very simple dictum to apply to the issue of missing data occurs when some of the variables are counts of events. In the case of relatively rare events, even in large datasets, it is not surprising to see counts of zero. For example, many people may have never been burgled or experienced a cardiac arrest. However, as argued in the earlier sections of this paper, in some cases non-reporting may depend on the circumstances of the potential reporter. If counts of burglaries are obtained from events processed by insurance companies, say at UK postcode level, those without insurance will not have their burglaries represented. However, choosing to be insured is not a purely random process, and non-insurance is generally a consequence of having a low income. Thus, the entries of zero in the database will likely be a mixture of those who have genuinely not been burgled and those who have been but were not insured. If the data were being used to assess the benefits of installing an alarm system, it is quite possible these would be underestimated—low-income households without insurance may well also not be able to afford home security systems, with data reflecting a “phantom” set of unprotected homes experiencing no burglaries. This example is essentially a special case of the big data paradox, but the focusing on counts of zero highlights an important issue: although some zeroes are genuine, other are the result of exclusion. In failing to count excluded events, and hence the people experiencing those events, extreme and problematic biases occur.

As in many situations, it is important to be aware on the entire process of what happens in the real world and what finally appears on the database. Rarely, if ever, does the final data perfectly reflect the phenomena that the researcher wishes to study. In the case of designed surveys and experiments, many actions are taken to make the data as faithful a representation of the reality being considered as possible. However, for most big data, far fewer precautions are taken, and the only realistic option is to be aware of any issues in the compiling and reporting of information and where possible to modify the analysis to take account of this. In the burglary example, a major issue is the overcount of zeros. Typically one might use a Poisson regression approach to analyze burglary counts, where a number of census variables may be investigated to see how strongly they associate with burglary counts. However a Poisson (or possibly negative binomial) model would presume that all zero counts are genuine. A *zero-inflated Poisson* model^6^ has an extra parameter allowing for a disproportionate number of zero counts:

(Equation 3) $$\left\{ \begin{array}{c} \mathrm{Pr}\left(y=0\right)=\pi +\left(1-\pi \right)e^{-\lambda }\\ \;\\ \mathrm{Pr}\left(y=m\right)=\left(1-\pi \right)\frac{e^{-\lambda }\lambda^{m}}{m!},\;m=1,2,3,\cdots \end{array}\right.$$

where π is the probability of a zero by exogenous exclusion, and λ is the mean of the underlying Poisson distribution. Using software such as *Stan*^7^ one can calibrate models such as this, where not only is λ linked to explanatory variables but also π, so that the extent of zero inflation can be modeled as a function of variables found in the big data source. In the burglary example, census-based indicators of deprivation could be used. Although this may only go partway to addressing this issue, it allows investigation into the existence of such a problem as well as offering some insight into potential factors leading to zeros arising from non-participation.

### Absence of evidence and evidence of absence

While zero as an observed count cannot be used as evidence of non-existence, one can argue that, for a near-population sample, it may provide stronger evidence for non-existence. Going back to our previous example, if no cases of burglary are reported by, say, 99% of the population, we might be more confident there are no cases as opposed to no burglaries reported by 10% of the population. Of course, in neither case can we conclude that there are no burglaries, but in the first case we can say it is at least rare (assuming the reporting of no burglaries is truthful). Assume a large sample, say near population size, report zero burglaries and only very small portion remain silent. In this case, one can assume the chance of having no burglaries in this city is very large.

In general, if no report/estimate is found for a large sample, the length of the confidence interval around zero will be smaller, inversely proportional to *N*. And as the sample size becomes bigger and bigger (e.g. near population), the confidence interval becomes smaller and smaller, around zero. For big data, the rule of three by Hanley and Lippman-Hand^8^ can be applied to have a better estimate to what degree “absence of evidence can be used as an evidence of absence.” The rule of three states if a certain event does not occur in a sample with n reports, the interval from 0 to 3/*n* is a 95% confidence interval for the rate of occurrences in the population of *N*.

However, there are several assumptions for the rule of three that may not be true in many cases. To be able to rely on the rule of three, we need to consider the effective sample size. It is also important to remember that rule of three is just an estimate for the probability estimate.

### Ethics of data we do not have

Although inferring patterns from missing data, imputing values for non-responded questions, and modeling the response behavior from missing data patterns are useful to understand the underlying reasons for having under-representations in the first place and ultimately designing services that can accommodate the needs and preferences of all, the main assumption here is “we can treat missing data as data.” Regardless of the accuracy and reliability of the output of the analysis, there is an ethical question. Missing data are the data that users intentionally or mistakenly have not shared; there is no consent to use of the data that we were not given. The goal of using missing data as data is to understand why missingness happens and what is the best estimate of the missing value. All of this might be in contrast with the agency and control of the individuals over their data. On the other hand, one can argue treating data that are not given as public property is not entirely wrong as nobody can bridge the confidentiality of something that has not been shared or even protected by the user. There is no clear conclusion to this debate, but certainly, there is a need for more study and investigation.
